# Reversing multidrug resistance in hepatocellular carcinoma cells by inhibiting extracellular signal-regulated kinase/mitogen-activated protein kinase signaling pathway activity

**DOI:** 10.3892/ol.2014.2521

**Published:** 2014-09-10

**Authors:** SIYUAN CHEN, YALI WANG, WENWEN RUAN, XIAOMIN WANG, CHAO PAN

**Affiliations:** 1 Department of Pathology, Zhongshan Hospital, Xiamen University, Xiamen, Fujian 361004, P.R. China; 2 Department of Hepatobiliary Surgery, Digestive Diseases Institute, Zhongshan Hospital, Xiamen University, Xiamen, Fujian 361004, P.R. China

**Keywords:** hepatocellular carcinoma, multidrug resistance, reverse, extracellular signal-regulated kinase/mitogen-activated protein kinase signaling pathway

## Abstract

The aim of the present study was to evaluate whether downregulation of extracellular signal-regulated kinase 1/2 (ERK1/2) is involved in conventional reversal methods and whether the inhibitors of the ERK signaling pathway reverse multidrug resistance (MDR) in hepatocellular carcinoma (HCC) cells. The sensitivities of SMMC7721 and BEL7402, and the MDR SMMC7721/Adriamycin (ADM) and BEL7402/ADM HCC cell lines to ADM were evaluated by CellTiter-Glo^®^ luminescent cell viability assay through calculating the half maximal inhibitory concentration (IC_50_) of ADM. In addition, the expression levels of ERK1/2 and phosphorylated (p)ERK1/2 were determined by western blot analysis subsequent to treatment of the cells with PD98059, an MEK inhibitor, or sorafenib, a multikinase inhibitor. The results revealed that the ADM IC_50_ for the SMMC7721/ADM cells was 16.44 times higher than that of the SMMC7721 cells (P<0.05), and the ADM IC_50_ for the BEL7402/ADM cells was 20.34 times higher than that of the BEL7402 cells (P<0.05). Following treatment with PD98059 or sorafenib, the expression levels of pERK1/2 in the MDR cells decreased in a dose-dependent manner. Subsequent to treatment with 5 μM PD98059, the ADM IC_50_ values for the SMMC7721/ADM and BEL7402/ADM cells were reduced to 0.8±0.056 and 1.583±0.284 μg/ml, respectively. Following treatment with 2.5 μM sorafenib, the ADM IC_50_ values for the SMMC7721/ADM and BEL7402/ADM cells were reduced to 0.264±0.049 and 1.099±0.135 μg/ml, respectively. Subsequent to incubation with 4 μg/ml cyclosporine A (CsA), a classic MDR reversal agent, the ADM IC_50_ values in the SMMC7721/ADM and BEL7402/ADM cells were reduced to 0.349±0.023 and 0.427±0.039 μg/ml, respectively. CsA treatment also increased the expression levels of pERK1/2 without affecting the total ERK1/2 levels. Therefore, the inhibition of ERK signaling pathway activity may be an important method to reverse the MDR of HCC cells, but is not unique.

## Introduction

Hepatocellular carcinoma (HCC) is the third most common cause of cancer-related mortality worldwide, with high recurrence and a low five-year survival rate (1). Thus far, excision remains the most significant method in the comprehensive treatment of HCC. However, resection of the tumor using surgery is difficult in the majority of cases of HCC, since >80% patients are suffering from advanced or unresectable diseases at the final diagnosis (2,3). Even following successful resection, the recurrence may be as high as 50% after two years (4). Chemotherapy is used as an adjuvant post-operative treatment, with the aim of reducing tumor recurrence. However, conventional systemic chemotherapy has shown only minor effectiveness with response rates of <10%, due to the intrinsic or acquired drug resistance caused by multidrug resistance (MDR) (4,5). The mechanisms of drug resistance are heterogeneous and include increased anticancer agent efflux by adenosine triphosphate (ATP)-binding cassette proteins, apoptotic inhibition, DNA repair activation and detoxifying system enhancement (6). Although numerous MDR reversal agents have been reported, the clinical application of these drugs is limited due to side-effects or toxicity that are unacceptable at the effective dose (7). Thus, identifying MDR reversal agents with high reversal activity and low toxicity is important.

Recently, evidence has accumulated demonstrating that the activation of the mitogen-activated protein kinase (MAPK) signaling pathway is associated with MDR in multiple types of tumors and that this signaling pathway has a predominant role in various cellular processes, including proliferation, differentiation, apoptosis, angiogenesis and migration (8–13). The MAPK group includes four distinct signaling cascades, which are known by the corresponding MAPK tier component: Extracellular signal-regulated kinase 1 and 2 (ERK1/2) (14–16); c-Jun N-terminal kinase 1 to 3 (17,18); p38 MAPK α, β, γ and δ (p38 α-δ) (19–22); and ERK5, also known as Big MAPK (23,24). The ERK1/2 cascade, which has been the most widely analyzed module in the MAPK signaling pathways, transmits predominantly mitogenic signals. The activation of the ERK1/2 signaling pathway is induced by guanosine 5′-triphosphate loading of Ras at the plasma membrane, which is followed by sequential activation of a series of protein kinases, including a member of the Raf family (such as Raf-1), MAPK or ERK kinase (MEK) 1/2, then ERK1 or ERK2 (25). Our previous study demonstrated that ERK1/2 is highly expressed in several HCC cells with MDR (26). The aim of the present study was to analyze whether the ERK/MAPK inhibitors reverse MDR in HCC cells. Furthermore, one classical MDR reversal agent, cyclosporine A (CsA), was selected to examine whether downregulation of ERK/MAPK signaling pathway activity is involved in the reversal mechanism of traditional methods, such as the inhibition of p-gp.

## Materials and methods

### Compounds

Sorafenib (Nexavar, BAY 43-9006), a multikinase inhibitor [for vascular endothelial growth factor receptor, platelet-derived growth factor receptor and rapidly accelerated fibrosarcoma (RAF) kinases], was manufactured by Bayer Pharmaceuticals (West Haven, CT, USA). PD98059, a MEK inhibitor, was purchased from Cell Signaling Technology, Inc. (Beverly, MA, USA). CsA was obtained from Enzo Life Sciences, Inc. (Farmingdale, NY, USA). The compounds were dissolved in 100% dimethyl sulfoxide (DMSO; Sigma-Aldrich, St. Louis, MO, USA) and diluted with RPMI 1640 to obtain a final DMSO concentration of 0.1% for the *in vitro* experiments. DMSO was subsequently added to the cell cultures at 0.1% (v/v) as a solvent control.

### Cell lines and cell culture

The SMMC7721 and BEL7402 human HCC cell lines were purchased from the Institute of Biochemistry and Cell Biology, Shanghai Institutes for Biological Science, Chinese Academy of Sciences (Shanghai, China). The SMMC7721 and BEL7402 cells were cultured with RPMI-1640 (HyClone Laboratories, Inc., Logan, UT, USA). The medium was supplemented with 10% calf serum, 100 IU/ml penicillin and 100 μg/ml streptomycin (all HyClone), and maintained at 37°C in a humidified atmosphere containing 50 ml/l CO_2_ and 950 ml/l air. To establish SMMC7721/Adriamycin (ADM) and BEL7402/ADM MDR cells, ADM (Shanghai Shenggong Biological Engineering Co., Ltd., Shanghai, China) was added to SMMC7721 and BEL7402 cells, respectively, at increasing stepwise concentrations between 1 and 5 mg/l. Resistant cells were selected by removing the non-resistant dead cells. MDR was maintained by culturing the cells with 5 mg/l ADM; the MDR cells were termed the SMMC7721/ADM and BEL7402/ADM cells. This study was approved by the ethics committee of the Affiliated Hospital of Xiamen University (Xiamen, China).

### CellTiter-Glo^®^ luminescent cell viability assay

The cells were plated at 3,000 cells per well in 96-well microtiter plates and incubated overnight at 37°C in a humidified incubator containing 5% CO_2_. On the following days, the corresponding compounds were added to the wells and the cultures were incubated for an additional 48 h. To investigate the drug resistance of MDR cells, the parental cells and MDR cells were exposed to various concentrations of ADM (0, 0.25, 1, 4, 16 or 64 μg/ml). A combination of various concentrations of ADM (0, 0.25, 1, 4, 16 or 64 μg/ml) and sorafenib (2.5 μM), PD98059 (5 μM) or CsA (4 μg/ml) were added to the experimental groups. Cell viability was determined using the CellTiter-Glo luminescent cell viability kit from Promega Corporation (Madison, WI, USA) according to the manufacturer’s instructions. This method was based on the measurement of ATP production in the cells, proportional to the number of viable cells, detected by luciferin-luciferase reaction. The cell proliferation inhibition rate was calculated by the following formula: Cell proliferation inhibition rate = (1 − relative luminescence of the experimental group/relative luminescence of the control group) × 100. All experiments were repeated at least three times and the average values were used as the final results. The half maximal inhibitory concentration (IC_50_) value, which signifies 50% cell growth inhibition compared with the control, was calculated by non-linear regression analysis with GraphPad Prism version 5.0 software (GraphPad Software, Inc., San Diego, CA, USA), according to the results of at least three independent experiments, with four replicates of each cell line per experiment. The resistance index (RI) and reversal fold were calculated according to the following formulae: RI = (IC_50_ of MDR cells)/(IC_50_ of parental cells); and reversal fold = (IC_50_ of MDR cells)/(IC_50_ of MDR cells following reversal).

### Western blot analysis

The cells were cultured in culture medium until 60–70% confluence was reached. DMSO and PD98059 (2.5, 5, 10 or 20 μM) were added to the control and experimental groups, which were incubated for 1 h. Sorafenib (2.5, 5 or 10 μM) or CsA (0.25, 1, 4, or 16 μg/ml) were then added to the experimental groups, and DMSO was added to the control groups. The cells were then incubated for 24 h. Adherent cells were washed with cold phosphate-buffered saline and lysed directly in the dish for 20 min on ice with cell lysis buffer [containing 150 mmol/l NaCL, 50 mmol/l Tris-HCL (pH 7.4), 2 mmol/l EDTA, 1% NP-40, protease inhibitor cocktail and phosphatase inhibitor cocktail; Applygen Technologies Inc., Beijing, China]. The lysates were then incubated at 4°C for 20 min and centrifuged for 10 min at 12,000 × g. The protein levels in the extracts were quantified using a bicinchoninic acid assay (Pierce Biotechnology, Inc., Rockford, IL, USA). Subsequently, the protein was denatured in a lithium dodecyl sulfate sample buffer for 5 min at 105°C. Equal quantities of total protein (20 μg per lane) were resolved on 12% polyacrylamide gels using standard sodium dodecyl sulfate polyacrylamide gel electrophoresis and then transferred to a polyvinylidene difluoride membrane (0.45 μm, Millipore, Billerica, MA, USA). The membranes were blocked with 5% dry milk in Tris-buffered saline (TBS) containing 0.05% Tween-20 (TBST) for 1 h at room temperature and incubated overnight at 4°C with the following primary antibodies: monoclonal rabbit anti-human, -mouse, -rat, -hamster, -monkey, -mink, -D. melanogaster, -zebrafish, -bovine, -dog, -pig, -S. cerevisiae, phosphorylated (p)ERK1/2 (1:2,000; Thr202/Tyr204; Cell Signaling Technology, Inc.), polyclonal rabbit anti-human, -mouse, -rat, -equine, -canine, -bovine, -porcine and -avian, ERK1/2 (1:200; Santa Cruz Biotechnology, Inc., Santa Cruz, CA, USA), polyclonal rabbit anti-human, -mouse, -rat and -baboon, GAPDH (1:1,000; Epitomics, Inc., Burlingame, CA, USA**).** Following incubation with the respective primary antibodies, the membranes were washed three times for 5 min in TBST. The memebranes were then exposed to horseradish peroxidase-conjugated monoclonal goat anti-rabbit immunoglobulin G (1:1,000; Multi Sciences (Lianke) Biotech Co., Ltd., Hangzhou, China) for 1 h at room temperature. Following incubation with the secondary antibodies, the membranes were washed three times for 5 min in TBST. The signal was detected with an Enhanced Chemiluminesence Western Blotting Detection kit (Applygen Technologies Inc.). The results are presented as the ratio of the density of the target protein to that of GAPDH. Each experiment was repeated at least three times and the final results are shown as the mean values.

### Statistical analysis

Statistical analysis was performed using SPSS version 13.0 (SPSS, Inc., Chicago, IL, USA) and data are shown as the mean ± standard deviation. Student’s t-test and a one-way analysis of variance were used for the statistical analyses. P<0.05 was considered to indicate a statistically significant difference.

## Results

### SMMC7721/ADM and BEL7402/ADM cells exhibit stable drug resistance

**Figure 1:**
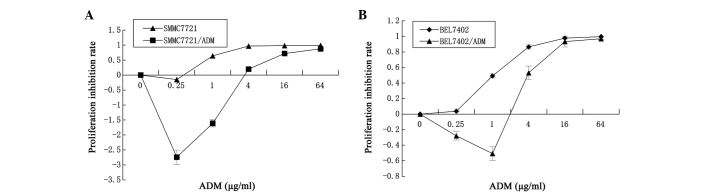
Increased drug resistance of SMMC7721/Adriamycin (ADM) and BEL7402/ADM cells to the cytotoxic drug, ADM, compared with that of SMMC7721 and BEL7402 cells. (A) The SMMC7721 and SMMC7721/ADM cells were incubated with 0, 0.25, 1, 4, 16 or 64 μg/ml ADM for 48 h. (B) The BEL7402 and BEL7402/ADM cells were also incubated with 0, 0.25, 1, 4, 16 or 64 μg/ml ADM for 48 h. At the end of incubation, the cell survival rates were determined by CellTiter-Glo^®^ luminescent cell viability assay and the proliferation inhibition rate was calculated. Results are reported as the mean ± standard deviation of three independent experiments performed in five replicates.

The ADM IC_50_ values of the SMMC7721 and SMMC7721/ADM cells were 0.089±0.026 and 1.463±0.168 μg/ml, respectively, and the RI of the SMMC7721/ADM cells was 16.44. The ADM IC_50_ values of the BEL7402 and BEL7402/ADM cells were 0.161±0.039 μg/ml and 3.266±0.271 μg/ml, respectively, and the RI of the BEL7402/ADM cells was 20.34. The results are shown in Fig. 1 and Table I. The data show that the ADM sensitivities of the SMMC7721/ADM and BEL7402/ADM cells were significantly lower than those of the corresponding non-resistant parent cells (P=0.000), which indicates that the SMMC7721/ADM and BEL7402/ADM cells exhibited stable chemoresistance.

### PD98059 and sorafenib inhibit ERK/MAPK signaling pathway activity in SMMC7721/ADM and BEL7402/ADM cells

**Figure 2:**
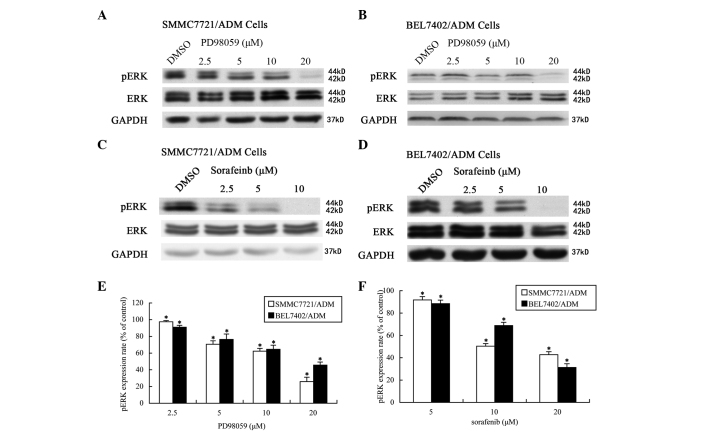
PD98059 and sorafenib inhibit the extracellular signal-regulated kinase (ERK) signaling pathway activity of SMMC7721/Adriamycin (ADM) and BEL7402/ADM multidrug-resistant cells. (A) SMMC7721/ADM and (B) BEL7402/ADM cells were treated with the indicated concentrations of PD98059 for 1 h. (C) SMMC7721/ADM and (D) BEL7402/ADM cells were treated with the indicated concentrations of sorafenib for 24 h. Following treatment, whole cell protein extracts were prepared. Western blot analysis was performed using specific antibodies against the indicated proteins. (E and F) The phosphorylated (p)ERK expression rate was calculated from the pERK density, and the rate in each control group was set as the 100% baseline. Columns represent the mean of three experiments with six samples in each group; bars indicate standard error. ^*^P<0.05 vs. control. DMSO, dimethyl sulfoxide.

Subsequent to 1 h of treatment with PD98059, the pERK1/2 expression rates (% of control) in the SMMC7721/ADM (Fig. 2A) and BEL7402/ADM (Fig. 2B) cells were downregulated in a dose-dependent manner. At concentrations of 2.5, 5, 10 and 20 μM, the rates declined to 97.43±1.51, 70.53±4.23, 62.33±3.34 and 25.79±5.33%, respectively, in the SMMC7721/ADM cells, and to 91.01±2.27, 86.31±6.54, 84.54±4.98 and 55.53±3.75%, respectively, in the BEL7402/ADM cells (Fig. 2E). Following 24 h of treatment with sorafenib at these same concentrations, pERK expression was again inhibited in a concentration-dependent manner (Fig. 2C and D). At concentrations of 2.5, 5 and 10 μM sorafenib, the pERK1/2 expression rates were reduced to 91.71±3, 50.41±2.3 and 42.76±2.6%, respectively, in the SMMC7721/ADM cells, and to 88.45±3.1, 68.79±2.9 and 31.28±3.3%, respectively, in the BEL7402/ADM cells (Fig. 2F).

### PD98059 and sorafenib increase the sensitivity of SMMC7721/ADM and BEL7402/ADM cells to ADM

**Figure 3:**
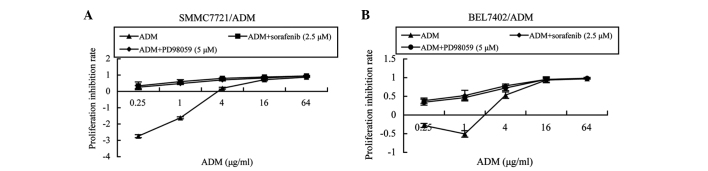
PD98059 and sorafenib enhance the sensitivity of SMMC7721/Adriamycin (ADM) and BEL7402/ADM cells to ADM. (A) PD98059 and sorafenib increased the cell proliferation inhibition induced by ADM in the SMMC7721/ADM cells. (B) PD98059 and sorafenib increased the cell proliferation inhibition induced by ADM in the BEL7402/ADM cells. The cell proliferation viability was detected by the CellTiter-Glo luminescent^®^ cell viability assay. Each value represents the average of four independent determinations with five replicates per experiment. Bars indicate standard error.

Subsequent to 72 h of treatment, sorafenib inhibited MDR cell proliferation in a dose-dependent manner. When the drug concentration was 2.5 μM, the cell inhibition rates were <5%, at 4.35 and 1.84% for the SMMC7721/ADM and BEL7402/ADM cells, respectively. Thus, 2.5 μM was selected to be the concentration of sorafenib used for MDR reversal. Similarly, when the concentration of PD98059 was 5 μM, the cell inhibition rates for SMMC7721/ADM and BEL7402/ADM cells were 3.78 and 1.21%, respectively; therefore, 5 μM was selected as the reversal concentration of PD98059. The cell proliferation inhibition rates in the cells treated with a combination of ADM plus PD98059 or sorafenib were higher than those treated with ADM only (Fig. 3A and B). When 5 μM PD98059 was added, the ADM IC_50_ values of the SMMC7721/ADM and BEL7402/ADM cells were 0.8±0.056 and 1.583±0.284 μg/ml, respectively. Furthermore, the reverse fold values were 1.83 in the SMMC7721/ADM cells and 2.06 in the BEL7402/ADM cells. When treated with 2.5 μM sorafenib, the ADM IC_50_ values of the SMMC7721/ADM and BEL7402/ADM cells were 0.264±0.049 and 1.099±0.135 μg/ml, respectively. The reversal fold ADM resistance levels of the SMMC7721/ADM and BEL7402/ADM cells were 5.54-fold and 2.97-fold, respectively (Table II).

### CsA enhances ADM sensitivity and upregulates ERK 1/2 phosphorylation in MDR HCC cells

**Figure 4:**
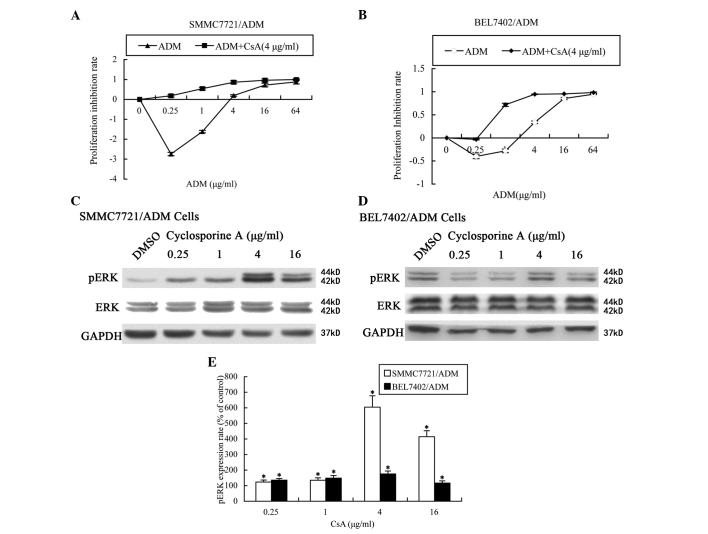
Cyclosporine A (CsA) enhances the sensitivity of cells to Adriamycin (ADM) and increases extracellular signal-regulated kinase (ERK)/mitogen-activated protein kinase signaling pathway activity in SMMC7721/ADM and BEL7402/ADM cells. (A and B) CsA increased the cell proliferation inhibition induced by ADM in the SMMC7721/ADM and BEL7402/ADM cells. The cell proliferation viability was detected by CellTiter-Glo^®^ luminescent cell viability assay. Each value represents the average of four independent determinations with five replicates per experiment. Bars indicate standard error. (C) SMMC7721/ADM and (D) BEL7402/ADM cells were treated with the indicated concentrations of CsA for 24 h. Following treatment, whole cell protein extracts were prepared. Western blot analysis was performed using specific antibodies against the indicated proteins. (E) The phosphorylated (p)ERK expression rates were calculated from the pERK density, and the rate in each control group was set as the 100% baseline. Columns represent the mean of three experiment with six samples in each group; bars indicate standard error. ^*^P<0.05 vs. control.

As shown in Fig. 4A and B, the cell proliferation inhibition rate of the cells cultured with ADM plus CsA was significantly increased compared with that of the cells cultured with ADM only (P=0.000). When combined with 4 μg/ml CsA, the ADM IC_50_ values of the SMMC7721/ADM and BEL7402/ADM cells were 0.349±0.023 and 0.427±0.039 μg/ml, respectively. The ADM resistance reversal levels of the SMMC7721/ADM and BEL7402/ADM cells were 4.19-fold and 7.65-fold, respectively (Table II). Following CsA treatment for 24 h, the pERK1/2 levels increased in a dose-dependent manner up to 4 μg/ml CsA and then declined at higher concentrations, but remained above the basal level. The total ERK1/2 levels were unchanged (Fig. 4C–E).

## Discussion

HCC is the third most common cause of cancer mortality, resulting in more than half a million fatalities annually worldwide (27,28). In addition to surgical intervention, systemic chemotherapy also has a significant role in HCC treatment, particularly for patients with advanced HCC (29). However, since traditional systemic chemotherapy has limited benefits in advanced-stage HCC patients due to MDR, novel approaches to overcome this resistance and offer patients tailored treatment strategies are urgently required (2,3). A number of MDR reversal agents have been developed, using the possible mechanisms being reported (30,31). However, toxicity has become the predominant obstacle in the wide application of these agents in clinical treatment (32). For instance, verapamil treatment induces cardiac toxicity and CsA exerts significant immunosuppressive effects and has renal toxicity. Therefore, identifying safe and efficient reversal agents is of vital importance for treating advanced-stage HCC patients.

The MAPK signaling pathway is known to mediate a number of cellular processes, including cell growth, differentiation, survival and apoptosis. Aside from these fundamental functions, MAPK has also been reported to be involved in the development of MDR, conferring imatinib resistance in acute lymphoblastic leukemia cells (33), vincristine resistance in gastric cancer cells (34) and anthracycline resistance in breast cancer cells (35).

Among the four MAPK signaling pathways, the ERK1/2 cascade is the most widely investigated. Thus far, the majority of studies have reported a positive correlation between the overactivation of ERK and the development of chemoresistance in numerous types of cancer cells (36–38). Certain studies have suggested that modulation of ERK activation may be a novel method in reversing MDR (40,41). Our previous study also demonstrated that ERK1/2 activity is upregulated in MDR HCC cells (26). As determined by these findings, inhibitors of the ERK/MAPK signaling pathway have been suggested to reverse MDR in HCC cells. In the present study, the upstream proteins, RAF and MEK, key proteins in the RAS/RAF/MEK/ERK cascade, were selected as the targets of inhibition. The results revealed that sorafenib and PD98059, which inhibit RAF and MEK kinases, respectively, downregulated the pERK1/2 levels without affecting the levels of total ERK1/2. The results of the cell viability assays demonstrated that sorafenib and PD98059 reduced the ADM IC_50_ values in the SMMC7721/ADM and BEL7402/ADM cells, indicating that the two inhibitors reverse the resistance of HCC cells to ADM. Together, these results suggest the possibility of using the inhibitors of the ERK/MAPK signaling pathway as MDR reversal agents, thus providing evidence for the use of these inhibitors in combination with traditional chemotherapeutic drugs in treating HCC patients.

CsA, an inhibitor first used to analyze MDR reversal, enhances the apoptosis in tumor cells induced by chemotherapeutics through increasing the intracellular drug concentration. The MDR reversal mechanism of CsA possibly occurs through inhibiting the pump function of p-gp (42,43). In the present study, CsA upregulated, but did not downregulate, the expression of pERK1/2 in the HCC MDR cells. These results indicate that the downregulation of pERK1/2 was not involved in the reversal function of CsA, suggesting that the inhibition of the ERK/MAPK signaling pathway is not the only method to reverse MDR in HCC cells. In addition to leukocytes, CsA exerts potent effects on a number of distinct types of cells and thus, regulates disparate biological functions (44–48). Furthermore, evidence is emerging that CsA regulates cell proliferation and invasion through ERK (44,49–51). Therefore, the increased pERK1/2 levels observed may be involved in certain other CsA effects in HCC cells. CsA treatment may therefore render cells in a state of stress, thus resulting in the upregulation of pERK1/2 through negative feedback.

The combined application of various chemotherapeutants is one method for mitigating drug resistance in classic chemotherapy (52). If the signaling pathway that exerts a predominant role in the growth tumor of a particular tumor becomes clear prior to the patient accepting therapy, the inhibitors of the corresponding signaling pathway may be pointedly selected to raise the effectiveness of chemotherapy. All results from the present study indicate that inhibition of ERK/MAPK signaling pathway activity may indeed reverse MDR in HCC cells, thus providing evidence for the use of ERK/MAPK signaling pathway inhibitors combined with traditional drugs in treating HCC. In addition, downregulation of the ERK/MAPK signaling pathway activity was not found to be involved in the CsA reversal function, which indicates that inhibiting ERK/MAPK signaling pathway activity is not a unique method to reverse MDR in HCC cells.

## Figures and Tables

**Table I:** ADM IC_50_ in SMMC7721, SMMC7721/ADM, BEL7402 and BEL7402/ADM cells.

Cell line	ADM IC_50_, μg/ml	Resistance index
SMMC7721	0.089±0.006	
SMMC7721/ADM	1.463±0.068	16.44^a^
BEL7402	0.161±0.03	
BEL7402/ADM	3.266±0.072	20.34^b^

a P<0.05 vs. SMMC7721;

b P<0.05 vs. BEL7402.

IC_50_, half maximal inhibitory concentration; ADM, Adriamycin.

**Table II:** ADM IC_50_ in hepatocellular carcinoma multidrug-resistant cells treated with PD98059, sorafenib and CsA.

Cell line/treatment	ADM IC_50_, μg/ml	Reverse-fold
SMMC7721/ADM	1.463±0.168	
SMMC7721/ADM+PD98059	0.800±0.056	1.83^a^
SMMC7721/ADM+sorafenib	0.264±0.049	5.54^a^
SMMC7721/ADM+CsA	0.349±0.023	4.19^a^
BEL7402/ADM	3.266±0.271	
BEL7402/ADM+PD98059	1.583±0.284	2.06^b^
BEL7402/ADM+sorafenib	1.099±0.135	2.97^b^
BEL7402/ADM+CsA	0.427±0.039	7.65^b^

a P<0.05 vs. SMMC7721/ADM;

b P<0.05 vs. BEL7402/ADM.

IC_50_, half maximal inhibitory concentration; ; CsA, cyclosporine A; ADM, Adriamycin.
